# Incidence and challenges of helicopter emergency medical service (HEMS) rescue missions with helicopter hoist operations: analysis of 11,228 daytime and nighttime missions in Switzerland

**DOI:** 10.1186/s13049-021-00898-y

**Published:** 2021-07-12

**Authors:** Urs Pietsch, Jürgen Knapp, Michael Mann, Lorenz Meuli, Volker Lischke, Mario Tissi, Stephen Sollid, Simon Rauch, Volker Wenzel, Stefan Becker, Roland Albrecht

**Affiliations:** 1 Department of Anaesthesiology and Intensive Care Medicine, Cantonal Hospital St. Gallen, Rorschacher Strasse 95, 9007 St. Gallen, Switzerland; 2 Air Zermatt, Emergency Medical Service, 3920 Zermatt, Switzerland; 3 Department of Emergency Medicine, Inselspital, Bern University, Bern, Switzerland; 4 Swiss Air-Ambulance, Rega (Rettungsflugwacht/Guarde Aérienne), Zurich, Switzerland; 5 Department of Anaesthesiology and Pain Medicine, Inselspital, Bern University Hospital, University of Bern, 3010 Bern, Switzerland; 6 Department of Vascular Surgery, University Hospital Zurich, Zurich, Switzerland; 7 Norwegian Air Ambulance Foundation, PB 414 Sentrum, 0103 Oslo, Norway; 8 University of Stavanger, PB 8600 Forus, 4036 Stavanger, Norway; 9 Institute of Mountain Emergency Medicine, Eurac Research, Bozen, Italy; 10 Department of Anaesthesiology and Intensive Care Medicine, F. Tappeiner Hospital, Merano, Italy; 11 Klinik für Anästhesie Klinikum Friedrichshafen GmbH, Röntgenstraße 2, 88048 Friedrichshafen, Germany

## Abstract

**Objective:**

We aimed to investigate the medical characteristics of helicopter hoist operations (HHO) in HEMS missions.

**Methods:**

We designed a retrospective study evaluating all HHO and other human external cargo (HEC) missions performed by Swiss Air-Rescue (Rega) between January 1, 2010, and December 31, 2019.

**Results:**

During the study period, 9,963 (88.7 %) HEMS missions with HHO and HEC were conducted during the day, and 1,265 (11.3 %) at night. Of the victims with time-critical injuries (NACA ≥ 4), 21.1 % (*n* = 400) reached the hospital within 60 min during the day, and 9.1 % (*n* = 18) at night. Nighttime missions, a trauma diagnosis, intubation on-site, and NACA Score ≥ 4 were independently and highly significantly associated with longer mission times (*p* < 0.001). The greatest proportion of patients who needed hoist or HEC operations in the course of the HEMS mission during the daytime sustained moderate injuries (NACA 3, *n* = 3,731, 37.5 %) while practicing recreational activities (*n* = 5,492, 55.1 %). In daytime HHO missions, the most common medical interventions performed were insertion of a peripheral intravenous access (*n* = 3,857, 38.7 %) and administration of analgesia (*n* = 3,121, 31.3 %).

**Conclusions:**

Nearly 20 % of patients who needed to be evacuated by a hoist were severely injured, and complex and lifesaving medical interventions were necessary before the HHO procedure. Therefore, only adequately trained and experienced medical crew members should accompany HHO missions.

## Introduction

Involvement of a helicopter emergency medical service (HEMS) can significantly shorten rescue times and improve the outcome of severely injured patients, especially in mountainous areas [1, 2]. Due to the challenging terrain in the mountains, landing a helicopter is not always possible, and alternative patient evacuation methods must be used. A helicopter hoist operation (HHO) is a common operational rescue technique used by mountain emergency medical services to extricate patients if landing at the scene of the incident is not possible. This helicopter procedure enables both immediate professional medical care onsite and a safe evacuation of the patient to ensure further outcome-relevant timely treatment at an appropriate hospital. A minority of all HHO missions take place during the night. Thus, there is less experience in night HHO missions per se.

In Switzerland and most European countries, the HEMS crew includes a pilot, a flight paramedic with additional training as a winch operator, and a physician. If the operation site is expected to be in challenging or exposed terrain, a mountain guide joins the team.

The deployment of an HEMS physician in the out-of-hospital setting is a controversial topic worldwide. The United States and other English-speaking countries typically provide paramedic-led prehospital trauma care, whereas in other predominantly European countries, emergency physicians are an integral part of prehospital HEMS [3, 4]. However, in recent years several countries (e.g., the United Kingdom, Norway, Finland) have introduced a supplementary physician-based model for advanced critical care out of hospital, and in particular for HEMS [4].

.The topic of HHO in HEMS missions is very rarely addressed in the scientific medical literature [3–5]. We do know that a reduction in visual cues at night poses an additional but manageable risk for HEMS operations.

The aim of this study was to investigate characteristics of HHO in HEMS, describing the severity of injuries and the type, frequency and timing of medical interventions.

## Methods

### Data and Ethics

We conducted a retrospective study of all consecutive HHO missions performed by Swiss Air-Rescue (Rega) from January 1, 2010, to December 31, 2019. Data were extracted from Rega’s HEMS staff mandatory electronic medical record system (SAP database), and an additional chart review was conducted. The cantonal ethics committee of St. Gallen (EKOS) reviewed the study design and granted permission for the use of patient data without individual patient consent according to the Federal Act on Research Involving Human Beings and the Ordinance on Human Research Except for Clinical Trials. The permission covers the processing of patient data from Rega’s HEMS operation (EKOS St. Gallen 10.2.2020, BASEC Nr. 2020 − 00252 EKOS 20/020).

### Setting and Population

In Switzerland, five organisations provide physician-staffed HEMS operations 24/7. About 2/3 of these are primary pre-hospital retrieval, and 1/3 provide secondary inter-hospital transfer. Rega is the largest of these organisations, with more than 88,000 HEMS missions in the observation period. Rega operates 12 helicopter bases and can reach any location in the operational area within 15 min of flight time day and night, provided the respective weather conditions are met. The helicopter fleet comprises seven Airbus H145 at the midland bases and 11 AgustaWestland AW109SP “Da Vinci” helicopters (performance-enhanced version for Rega of the AW109S Grand) at the alpine bases. More than 11,000 HEMS missions are conducted per year with Rega’s helicopters, and all are equipped with a certified rescue hoist and avionics that permit night operations with and without night vision goggles (NVG) under visual flight rules (VFR), but also under instrument flight rules (IFR).

In Switzerland, the HEMS crew includes a pilot, an HEMS physician, and a paramedic, who serves as technical crew member and hoist operator. Inter alia, the requirements for HEMS physicians are a board certification in anaesthesiology and a certification in pre-hospital emergency medicine. Several HEMS physicians hold additional certifications in intensive and critical care medicine and/or mountain emergency medicine. In missions, when challenging terrain is expected, a rescue specialist with basic life support education is added to the crew on board. The HEMS physician is either winched down to the site first or after the rescue specialist’s initial safety assessment of the environment and situational circumstances.

### Definitions and Statistics

For this study we analysed mission characteristics, including mission duration, time of day, season [6], the National Advisory Committee for Aeronautics score (NACA) [7], and the medical interventions performed on scene. That included vascular access, analgesia, immobilisation, CPR, and endotracheal intubation (either drug-assisted intubation (RSI) or intubation during cardiac arrest). Nighttime was defined according to the European Union Aviation Safety Agency (EASA) [8] as the period between the end of evening civil twilight and the beginning of morning civil twilight.

Continuous variables were summarised by mean ± standard deviation if normally distributed, or by median and interquartile range if skewed. Normality was tested using the Shapiro-Wilk test. Categorical variables were summarised with counts and percentages for each level of the variable. Changes in the number of missions per year were assessed by linear regression, and the total number of missions per base type was compared using Pearson’s Chi-squared test. The Wilcoxon-Mann-Whitney test was used to assess differences in the duration of daytime and nighttime HHO missions. To further investigate factors that are potentially associated with a prolonged duration of HHO missions (mission time was defined as the time between an emergency call and arrival at the hospital), a multivariable linear regression model was built including the binary variables intubation, daytime/nighttime, and trauma versus medical diagnosis as well as the NACA score as a factor variable. To obtain a more homogeneous sample, unharmed patients (NACA 0) were excluded from this analysis; minor and moderately injured patients (NACA 1–3) were merged; and deceased patients (NACA 7) were excluded, as there was not a clear end-of-mission time point defined for a substantial proportion of these patients. The resuscitation policy of Rega is to stay onsite until ROSC, with the exception of cardiac arrest in special circumstances (e.g., deep hypothermia or transport under CPR to a hospital). Two-sided p-values of < 0.05 were considered statistically significant. All statistical analyses were performed using R Studio 3.6.0 on macOS 10.15.4.

## Results

### Number of HHO Missions in Switzerland

During the study period, 88,213 HEMS missions were recorded, 11,228 of which were registered as HHO missions. The majority of HHO missions (9,963; 88.7 %) were conducted during the day. There were 1,265 (11.3 %) nighttime missions (Table 1), most of which took place before midnight (n = 1,050, 83 %). All patients were winched up accompanied by either a rescue specialist or the HEMS physician. All patients and rescuers safely boarded the helicopter, without any procedure-related injuries or other adverse events involving patients or crew members, as recorded for the observation period.

**Table 1:** Characteristics of HHO Missions (*n*=11,228)

**Variable**	Day *n*= 9,963 (88.7%)	Night *n*= 1,265 (11.3%)
**Age, mean years ± SD**	46.8 ±19.2	40.5 ±19.2
Neonate <1 day, n (%)	22 (0.2)	2 (0.2)
<18 years, n (%)	498 (5)	103 (8.1)
80+ years, n (%)	297 (3.0)	23 (1.8)
Unknown/Missing	57 (0.6)	12 (0.9)
**Accident Occurrence, n (%)**		
Hiking	3,568 (35.8)	411 (32.5)
Climbing / mountaineering	1,522 (15.3)	245 (19.4)
Paragliding	402 (4.0)	27 (2.1)
Winter sports (skiing, freeriding, etc.)	975 (9.8)	123 (9.7)
Road accident	1,388 (13.9)	56 (4.4)
Other	1,393 (14.0)	306 (24.2)
Unknown / missing	715 (7.2)	97 (7.7)
**NACA Score**^+^**, n (%)**		
0 = No injury or disease	2,259 (22.7)	610 (48.2)
1 = Injuries/diseases without any need for acute physician care	395 (4.0)	80 (6.3)
2 = Injuries/diseases requiring examination and therapy by a physician but hospital admission is not indicated	820 (8.2)	51 (4.0)
3 = Injuries/diseases without acute threat to life but requiring hospital admission	3,731 (37.5)	219 (17.3)
4 = Injuries/diseases that can possibly lead to deterioration of vital signs	1,466 (14.7)	149 (11.8)
5 = Injuries/diseases with acute threat to life	380 (3.8)	46 (3.2)
6 = Injuries/diseases requiring resuscitation	53 (0.5)	4 (0.3)
7 = Lethal injuries or diseases (with or without resuscitation attempts)	859 (8.6)	106 (8.4)
**Diagnosis, n (%)**		
Trauma	6,430 (64.5)	528 (41.7)
Medical	1,119 (11.2)	104 (8.2)
Uninjured	2,414 (24.2)	633 (50.0)
**Procedures Performed, n (%)**		
Analgesia	3,121 (31.3)	202 (16.0)
Peripheral vascular access	3,857 (38.7)	268 (21.2)
Endotracheal intubation	196 (2.0)	13 (1.0)
Cardiopulmonary resuscitation	176 (1.8)	11 (0.9)
Mucosal atomisation device	73 (0.7)	11 (0.9)
Immobilisation vacuum mattress	1,994 (20.0)	185 (14.6)
Needle thoracostomy	19 (0.2)	1 (0.1)
Surgical thoracostomy	8 (0.1)	1 (0.1)
**Mission Times, median minutes (IQR)**		
Emergency call to take-off at base	9 (7 to 15)	22 (11 to 36)
Take-off at base to hospital	52 (40 to 67)	69 (50 to 94)
Emergency call to hospital	67 (54 to 83)	83 (73 to 129)
**Reached the hospital ≤60 min**		
Overall	2,668 (26.8)	71 (5.6)
NACA 1 - 3 (Day: *n* = 4946; Night: *n* = 350)	2,206 (44.6)	43 (12.3)
NACA 4 - 6 (Day: *n* = 1899; Night: *n* = 199)	400 (21.1)	18 (9.1)

Data were complete if not otherwise stated. *SD* standard deviation; *NACA* National Advisory Committee for Aeronautics; +Trauma patients were in significantly worse condition than non-trauma patients (*p* < 0.001)  in terms of NACA score.

### Regional Distribution

**Fig. 1:**
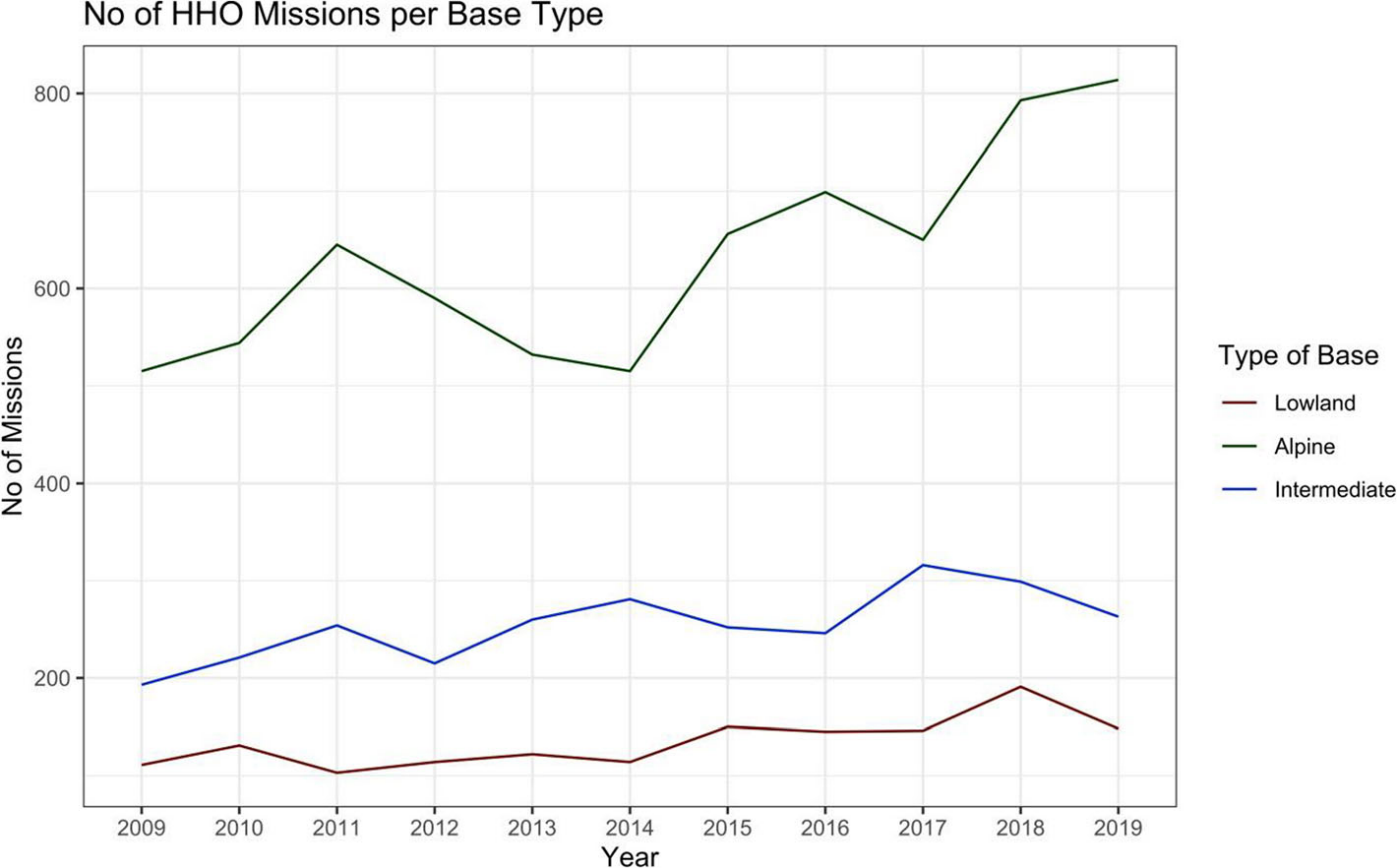
Total Number of HHO Missions per Base Type during the Study Period. No missing data. *P*-values for slope within each group calculated with linear regression models

**Fig. 2:**
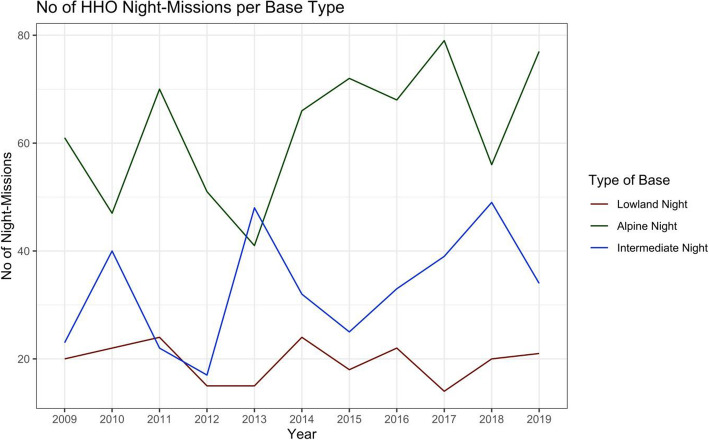
Number of Nighttime HHO Missions per Base Type during the Study Period. No missing data. *P*-values for slope within each group calculated with linear regression models

There was a significant increase in the total number of HHO missions over the study period for all types of HEMS bases (i.e., lowland, intermediate, alpine; Fig. 1). The number of HHO night missions did not significantly increase over the study period (Fig. 2), but there was a positive tendency (i.e., positive regression coefficients for alpine and intermediate bases). The total number of HHO missions and the number of nighttime HHO missions were significantly higher for alpine bases compared to intermediate or lowland bases (*p* < 0.001 for both comparisons).

### Mission Duration

**Fig. 3:**
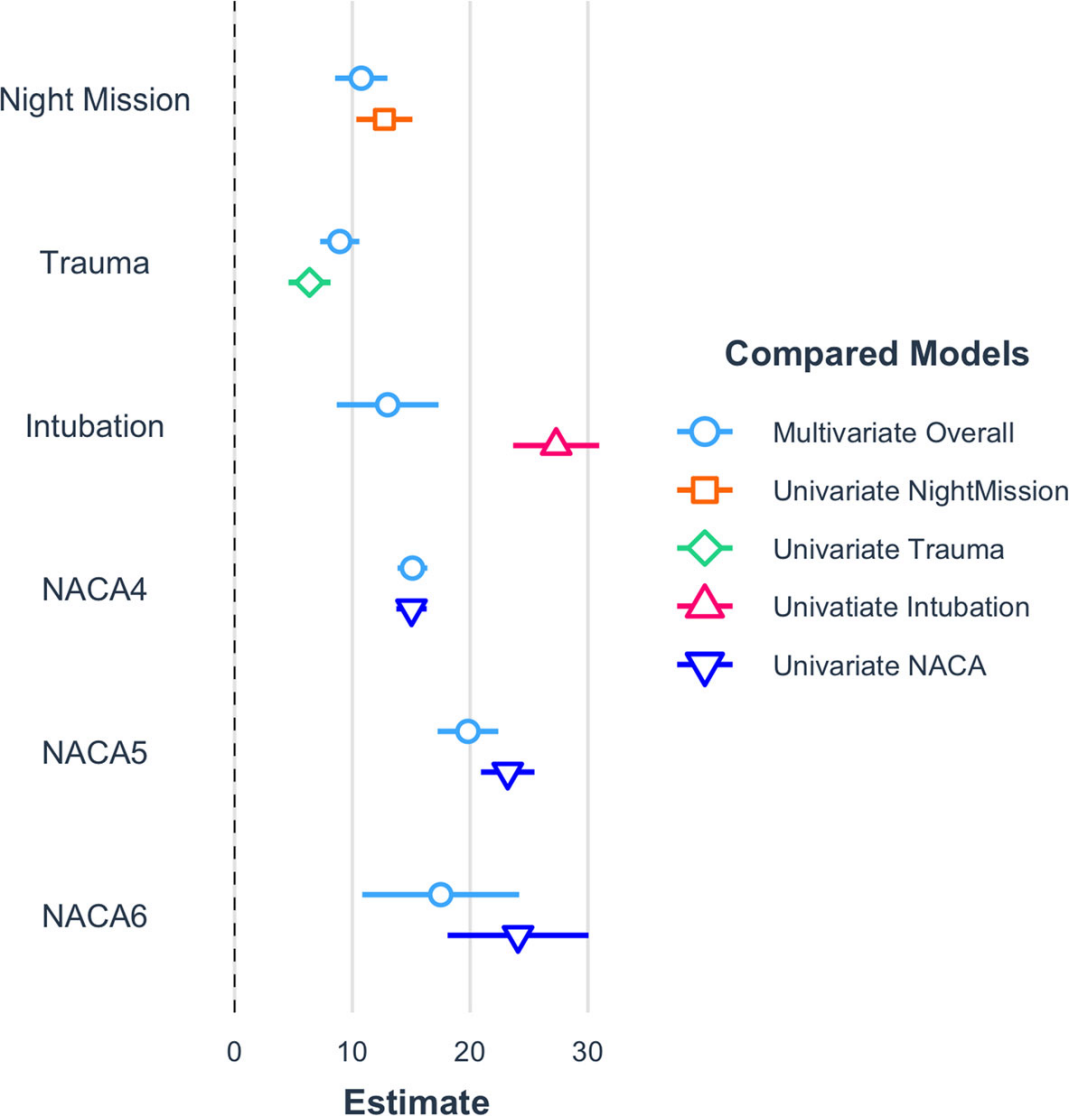
Linear regression models on duration of HHO-Missions. Visualisation of the multivariable and univariate linear regression models as presented in Table 2. Estimates are in minutes. Reading Example: Intubation prolonged the total mission time by 27 min. (95 % CI 24–31 min.) in the univariate analysis; adjusted for trauma, nighttime mission and NACA score intubation prolonged the overall mission time by 13 min. (9–17 min.)

The overall median time from emergency call to landing at the hospital in an HHO mission was significantly shorter during the day compared to at night as well (67 min; IQR 54 to 83 min versus 83 min; IQR 73 to 129 min, *p* < 0.001) (Table 1). Victims with possibly life-threatening injuries (NACA ≥ 4), 21.1 %; *n* = 400) reached the hospital within 60 min in the daytime, and 9.1 % of patients (n = 18) reached the hospital within 60 min during the night. A trauma diagnosis, night missions, intubation on-site, and NACA Score ≥ 4 were independently and highly significantly associated with longer mission times (*p* < 0.001 for all variables in univariate and multivariate analysis) (Table 2; Fig. 3). In the univariate analysis, intubation prolonged the overall mission time by roughly 27 min. Multivariate analysis revealed that intubation itself is only accountable for an additional 13 min when adjusted for trauma diagnosis, night mission, and NACA score. The other variables (night mission, trauma diagnosis, and NACA score) were less affected by the multivariate adjustments.

**Table 2:** Linear Regression Models on Duration of HHO Missions

Variable	Multivariate Adjusted			Univariate Analysis		
	**Estimate**	**95 % -C.I.**	***p*-value**	**Estimate**	**95 % - C.I.**	***p*-value**
Night mission	10.76	8.54 to 12.98	< 0.001	12.72	10.34 to 15.10	< 0.001
Trauma	8.93	7.26 to 10.60	< 0.001	6.36	4.58 to 8.15	< 0.001
Intubation	13.00	8.68 to 17.32	< 0.001	27.30	23.65 to 30.96	< 0.001
NACA 4	15.10	13.84 to 16.37	< 0.001	15.02	13.74 to 16.30	< 0.001
NACA 5	19.81	17.24 to 22.39	< 0.001	23.19	20.92 to 25.46	< 0.001
NACA 6	17.50	10.84 to 24.16	< 0.001	24.06	18.08 to 30.05	< 0.001

Complete case analysis of 6,427 patients (excluded from the analysis were patients with NACA 0 and 7). Unit of estimates: minutes. Fit of the multivariate model: p < 0.001, Adj. R = 0.148. Night mission: day missions served as the reference group

Trauma: Medical diagnosis served as the reference group. *NACA* NACA was analysed as a factor variable, scores 1–3 were merged and served as the reference group

### Characteristics of HHO Missions and Medical Condition of Patients

About half of the HHO missions taking place at night in this study were performed for uninjured patients (NACA 0, n = 610, 48.2 %), whereas during the daytime only 22.7 % (n = 2,259) were uninjured. Most HHO missions during the daytime were due to winter or summer sport-related injuries of moderate severity (NACA 3, n = 3,731, 37.5 %). The greatest proportion of patients requiring hoist operations during the daytime were practicing recreational activities in the mountains during the summer (hiking, mountaineering, climbing, etc.) (n = 5,492, 55.1 %), whereas 975 patients (9.8 %) were doing winter sports such as skiing, snowboarding or free riding. Road accidents accounted for 1,388 (14.0 %) evacuations by HHO (Table 1). A substantial number of patients in HEMS missions with HHO during day or night were dead on arrival on scene or died on scene (NACA 7, n = 859, 8.6 %, and n = 106, 8.4 %, respectively). Return of spontaneous circulation (ROSC) could be achieved in 5 % (n = 56) of patients with cardiac arrest. Trauma victims were in significantly worse condition according to the NACA score compared to medical patients (*p* < 0.001, Chi2-test).

### Medical Interventions during HHO Missions

In daytime HHO missions, the most common interventions performed onsite before evacuation were insertion of peripheral intravenous access (n = 3,857, 38.7 %) and analgesia (n = 3,121, 31.3 %). Cardiopulmonary resuscitation was performed in 176 patients (1.8 %), 196 patients (2.0 %) were endotracheally intubated, and in 27 patients (0.3 %) chest decompression was performed (Table 1). Insertion of intravenous access (n = 268, 21.2 %) and analgesia (n = 202, 16.0 %) were also the most common procedures recorded at night. Only 11 patients (0.9 %) at night needed cardiopulmonary resuscitation, 13 patients (1.0 %) were intubated, and in 2 patients (0.2 %) chest decompression was performed (Table 1).

## Discussion

This study of 11,228 HHO rescue missions performed day and night is the largest known study to date. Our data show that HHO missions in Switzerland occur frequently, even at night. Although most of the patients evacuated by HHO had no or minor injuries, almost one fifth were in severe condition, with NACA scores between 4 and 6, and in many cases advanced medical interventions were performed at the scene before HHO evacuation. Night missions, a trauma diagnosis, intubation on-site, as well as NACA Score ≥ 4 were independently and highly significantly associated with longer mission duration. Nevertheless, the aforementioned factors increased the mission time in general, regardless of whether a hoist was employed. With regard to Rega’s additional safety procedures for night flight operations, there is a natural increase in the mission time devoted to flight and patient safety, and among other things address the operational risks resulting from the lack of daylight and the subsequent natural deficiencies of the human eye in dark environments. Flight and patient safety must never be compromised. Thus, compromising safety to save 2 or maybe 4 min is not an eligible or recommendable option.

### Need for HEMS Crews with Advanced Skills

A relevant observation in our study is that the proportion of severely injured patients (NACA 4–6) is similar in daytime and nighttime HHO missions. This emphasises the need for HEMS teams with advanced critical care capabilities 24/7, and adds weight to the discussion of personal skills in HEMS services [9]. In our study, the condition of trauma vs. medical victims was more critical judged by the NACA score (p < 0.001). This finding contradicts a previous Swiss study which showed that in HEMS, patients with medical emergencies had higher NACA scores than trauma patients [10, 11]. A possible explanation is an overall predominance of trauma in our study population, due to the fact that the greatest proportion of patients in need of HHO rescue are practicing recreational activities in the mountains.

We found that most of the basic medical interventions we provide— such as vascular access (n = 4,125; 36.8 %), analgesia (n = 3,323; 29.6 %) and immobilisation (n = 2,179; 19.4 %)—were performed on the scene and before the HHO procedure. In 425 patients (3.8 %), advanced critical care interventions (cardiopulmonary resuscitation, ventilation, rapid sequence induction, endotracheal intubation, pleural decompression) were performed urgently due to immediate life-threatening conditions such as cardiac arrest, acute respiratory failure, cardiocirculatory collapse, or pneumothorax. These findings are in accordance with previous reports and again emphasise that the medical team involved in the HHO rescue missions should be able to perform the entire spectrum of life-saving emergency procedures in often extremely difficult environmental conditions, and with limited personnel resources [12, 13]. Recent studies have shown a clear benefit for the survival of severely injured patients when an EMS team including a physician delivers prehospital care on site, compared to a “scoop and run” approach [2, 9, 14]. In Europe, primarily anaesthesiology and intensive care medicine physicians have the experience needed to perform these invasive procedures safely [15]. Health systems in other countries may have different legal settings that render other specialties or professions more relevant for HEMS staffing, but the goal should always be to provide the highest level of care possible.

There are some studies analysing prehospital times in alpine HEMS [5, 16, 17]. The mean overall prehospital time of alpine HEMS missions for severely injured trauma patients was found to be nearly two hours [18]. Analysis of our missions showed a mean prehospital time for HHO missions of 67 min during the daytime and 83 min at night. We found some factors that significantly prolong prehospital times, such as on-scene intubation, injury severity (NACA ≥ 4) and rescue of trauma patients as compared to non-trauma patients. Yet, overall, HHO do not seem to prolong the prehospital times when compared to alpine HEMS missions without HHO [16, 17, 19, 20].

We found a significantly higher proportion of HHO missions in the alpine HEMS compared to the intermediate and lowland bases. Additionally, there was a tendency towards an increase in HHO mission volume over the 10-year study period in the alpine HEMS bases. Both findings could be connected to an increase in recreational activities in the mountains, and more extreme and more remote leisure behaviour over time.

## Strengths

This is the first study analysing > 10,000 HHO missions including data of night missions [3, 11, 18].

## Limitations

Our study has limitations inherent in a retrospective chart review, as data quality depends on documentation quality. Second, we were unable to validate the pre-hospital diagnosis made by the HEMS team, or to determine in-hospital outcome because of the lack of related hospital follow-up in our database. Finally, composition of HEMS crews and legal aspects elsewhere may have an impact as well.

## Conclusions

Nearly 20 % of patients who needed to be evacuated by a hoist were severely injured, and complex and lifesaving medical interventions were necessary before the HHO procedure. Therefore, only adequately trained and experienced medical crew members should accompany HHO missions.

## Data Availability

Please contact author for data requests.

## Abbreviations

HEC: Human external cargo
HEMS: Helicopter emergency medical service
HHO: Helicopter hoist operation
NACA: National Advisory Committee for Aeronautics
RSI: Rapid sequence intubation
